# Intracranial Hemorrhage Complicating Acute Myocardial Infarction: An 18-Year National Study of Temporal Trends, Predictors, and Outcomes

**DOI:** 10.3390/jcm9092717

**Published:** 2020-08-22

**Authors:** Sri Harsha Patlolla, Pranathi R. Sundaragiri, Wisit Cheungpasitporn, Rajkumar Doshi, Gregory W. Barsness, Alejandro A. Rabinstein, Allan S. Jaffe, Saraschandra Vallabhajosyula

**Affiliations:** 1 Department of Cardiovascular Surgery, Mayo Clinic, Rochester, MN 55905, USA; patlolla.sriharsha@mayo.edu; 2 Division of Hospital Internal Medicine, Department of Medicine, Mayo Clinic, Rochester, MN 55905, USA; drpranathi99@gmail.com; 3 Division of Nephrology, Department of Medicine, School of Medicine, University of Mississippi, Jackson, MS 39216, USA; wcheungpasitporn@gmail.com; 4 Department of Medicine, Reno School of Medicine, University of Nevada, Reno, NV 89557, USA; raj20490@gmail.com; 5 Department of Cardiovascular Medicine, Mayo Clinic, Rochester, MN 55905, USA; barsness.gregory@mayo.edu (G.W.B.); jaffe.allan@mayo.edu (A.S.J.); 6 Division of Neurocritical Care and Hospital Neurology, Department of Neurology, Mayo Clinic, Rochester, MN 55905, USA; rabinstein.alejandro@mayo.edu; 7 Division of Pulmonary and Critical Care Medicine, Department of Medicine, Mayo Clinic, Rochester, MN 55905, USA; 8 Center for Clinical and Translational Science, Mayo Clinic Graduate School of Biomedical Sciences, Rochester, MN 55905, USA; 9 Section of Interventional Cardiology, Division of Cardiovascular Medicine, Department of Medicine, Emory University School of Medicine, Atlanta, GA 30322, USA

**Keywords:** intracranial hemorrhage, acute myocardial infarction, cerebrovascular circulation, complications, outcomes research

## Abstract

Background: There is a paucity of contemporary data on the burden of intracranial hemorrhage (ICH) complicating acute myocardial infarction (AMI). This study sought to evaluate the temporal trends, predictors, and outcomes of ICH in AMI. Methods: The National Inpatient Sample (2000–2017) was used to identify adult (>18 years) AMI admissions with ICH. In-hospital mortality, hospitalization costs, length of stay, and measure of functional ability were the outcomes of interest. The discharge destination along with use of tracheostomy and percutaneous endoscopic gastrostomy were used to estimate functional burden. Results: Of a total 11,622,528 AMI admissions, 23,422 (0.2%) had concomitant ICH. Compared to those without, the ICH cohort was on average older, female, of non-White race, had greater comorbidities, and had higher rates of arrhythmias (all *p* < 0.001). Female sex, non-White race, ST-segment elevation AMI presentation, use of fibrinolytics, mechanical circulatory support, and invasive mechanical ventilation were identified as individual predictors of ICH. The AMI admissions with ICH received less frequent coronary angiography (46.9% vs. 63.8%), percutaneous coronary intervention (22.7% vs. 41.8%), and coronary artery bypass grafting (5.4% vs. 9.2%), as compared to those without (*p* < 0.001). ICH was associated with a significantly higher in-hospital mortality (41.4% vs. 6.1%; adjusted OR 5.65 (95% CI 5.47–5.84); *p* < 0.001), longer hospital length of stay, higher hospitalization costs, and greater use of percutaneous endoscopic gastrostomy (all *p* < 0.001). Among ICH survivors (*N* = 13, 689), 81.3% had a poor functional outcome at discharge. Conclusions: ICH causes a substantial burden in AMI due to associated higher in-hospital mortality and poor functional outcomes.

## 1. Introduction

Intracranial hemorrhage (ICH) is a rare but catastrophic complication in patients with acute myocardial infarction (AMI) [1,2]. However, in the contemporary era, primary percutaneous coronary intervention (PCI) is being increasingly used over thrombolytic therapy for early revascularization, resulting in a decline in the incidence and burden of ICH after AMI [3,4,5,6]. Due to the uncommon occurrence of this complication in recent times, studies evaluating these patients are limited by the small number of ICH events [3]. Prior studies have often combined ICH patients with those with acute ischemic stroke, which limits individual assessments [5,6]. Concomitant ICH in AMI is associated with higher in-hospital mortality [5,7,8]. Importantly, patients with ICH that survive the index hospitalization tend to have a varying degree of disability, which has important implications on their health-related outcomes [9,10]. Contemporary data on the burden of ICH in AMI patients is limited. More importantly, very few large studies have reported functional outcomes of patients after ICH. Therefore, we used a large nationally representative database to evaluate the prevalence and predictors of ICH after AMI and the impact of ICH on the mortality and the functional outcomes of these patients.

## 2. Methods

The national (nationwide) inpatient sample (NIS) is the largest all-payer database of hospital inpatient stays in the United States and contains discharge data from a 20% stratified sample of community hospitals with information regarding each discharge [11]. Information regarding each discharge includes patient demographics, primary payer, hospital characteristics, principal diagnosis, up to 29 secondary diagnoses, and procedural diagnoses. Using the healthcare cost and utilization project (HCUP)-NIS data from 1 January 2000 through 31 December 2017, a cohort of adult admissions (>18 years) with AMI in the primary diagnosis field (International Classification of Diseases 9.0 Clinical Modification (ICD-9CM) 410.x and International Classification of Diseases 10 Clinical Modification (ICD-10CM) I21.x -22.x) were identified [12,13]. A concomitant diagnosis of ICH was identified using ICD-9CM 430, 431, 432.0, 432.9, and ICD-10CM I60x-62.x [14]. The Deyo’s modification of the Charlson Comorbidity Index was used to identify the burden of co-morbid diseases [15]. Patient demographic characteristics, procedural and in-hospital characteristics, and outcomes were identified for all admissions using previously used methodologies from our group and others (Supplementary Tables S1 and S2.) [12,13,16,17]. In ICH, discharge destination was previously reported to be strongly correlated to measure of disability in these admissions [9,10]. Prior studies classified clinical functional outcomes as good (none to minimal disability), defined as discharge to self-care with or without home health services; and poor (moderate to severe disability), defined as discharge to nursing facility, extended care facility, or hospice, or placement of a tracheostomy and/or gastrostomy. This has shown very good correlation with the modified Rankin scale [9].

The primary outcome of interest was the in-hospital mortality of AMI admissions with and without concomitant ICH. Hospitalization costs, hospital length of stay, and functional outcomes were also evaluated. Temporal trends of in-hospital mortality and poor functional outcomes in ICH survivors stratified by type of AMI were evaluated.

### Statistical Analysis

In accordance with HCUP-NIS recommendations, survey procedures using discharge weights and trend weights for 2000–2011 provided with the HCUP-NIS database were used to generate national estimates [18]. Chi-square and t-tests were used to compare categorical and continuous variables, respectively. Multivariable logistic regression was used to analyze trends of prevalence and in-hospital mortality over time (referent year 2000), and predictors of ICH with relevant baseline and in-hospital variables. Temporal trends in the proportion of poor functional outcomes in AMI-ICH admissions and in-hospital mortality were plotted using adjusted odds ratio relative to the referent year (2000) that were calculated using multivariable logistic regression analysis. For the multivariable modeling, regression analysis with purposeful selection of statistically (liberal threshold of *p* < 0.20 in univariate analysis) and clinically relevant variables was conducted. Two-tailed *p* < 0.05 was considered statistically significant.

## 3. Results

Between 1 January 2000 and 31 December 2017, there were a total of 11,622,528 admissions for AMI, of which ICH was noted in 23,422 (0.2%). The prevalence of ICH was slightly higher in those with ST-segment elevation AMI (STEMI) than in those with non-ST-segment elevation AMI (NSTEMI) (0.3% vs. 0.2%, *p* < 0.001). Compared to AMI admissions without ICH, those who had ICH were on average older, female, of non-White race, Medicare insured, had greater comorbidities, and had higher proportions of atrial and ventricular arrhythmias (Table 1). Higher proportions of concomitant acute organ failure, cardiac arrest, and cardiogenic shock were seen in AMI admissions complicated by ICH. Fibrinolysis was used more frequently (8.9% vs. 2.2%), whereas coronary angiography (40.5% vs. 63.6%), PCI (25.4% vs. 41.5%), and coronary artery bypass grafting (CABG) (5.4% vs. 9.2%) were used less frequently in the ICH cohort (all *p* < 0.001). In a multivariable logistic regression analysis, female sex, non-White race, higher comorbidity, STEMI presentation, acute organ failure, atrial fibrillation/flutter, use of fibrinolytic therapy, and mechanical circulatory support were identified as individual associations of ICH after AMI (Table 2).

**Figure 1:**
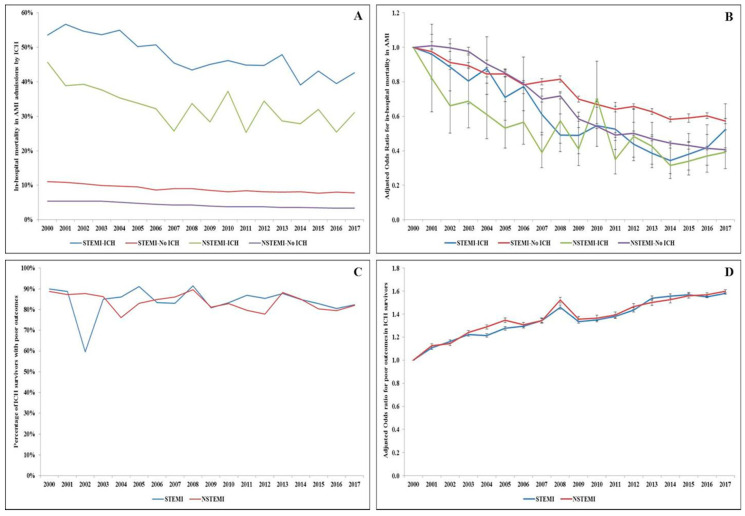
Trends in in-hospital mortality and poor functional outcomes in AMI admissions with and without ICH. (**A**) Unadjusted in-hospital mortality in AMI admissions stratified by type of AMI and the presence of ICH (*p* < 0.001 for trend over time); (**B**) adjusted odds ratio for in-hospital mortality by year (with 2000 as the referent) in AMI admissions stratified by type of AMI and the presence of ICH, adjusted for age, sex, race, comorbidity, primary payer, hospital region, hospital location and teaching status, hospital bed size, acute organ failure, cardiogenic shock, atrial fibrillation/flutter, cardiac arrest, coronary angiography, percutaneous coronary intervention, coronary artery bypass grafting, pulmonary artery catheterization, usage of fibrinolytic, vascular complications, mechanical circulatory support, invasive mechanical ventilation, and acute hemodialysis (*p* < 0.001 for trend over time); (**C**) Unadjusted temporal trends of the proportion of AMI-ICH admissions with poor functional outcome stratified by type of AMI (*p* < 0.001 for trend over time); (**D**) Adjusted odds ratio for poor functional outcome in STEMI and NSTEMI admissions by year (with 2000 as the referent); adjusted for age, sex, race, comorbidity, primary payer, hospital region, hospital location and teaching status, and hospital bed size, acute organ failure, atrial fibrillation/flutter, cardiogenic shock, cardiac arrest, coronary angiography, percutaneous coronary intervention, coronary artery bypass grafting, pulmonary artery catheterization, mechanical circulatory support, invasive mechanical ventilation, and acute hemodialysis (*p* < 0.001 for trend over time).

AMI admissions complicated by ICH had a significantly higher all-cause in-hospital mortality (41.4% vs. 6.1%; unadjusted odds ratio 10.86 (95% confidence interval 10.58–11.15); adjusted odds ratio 5.65 (95% confidence interval 5.47–5.84); *p* < 0.001) (Supplementary Table S2). There was a steady decrease in the adjusted in-hospital mortality in AMI admissions with and without ICH during the study period (Figure 1A,B). The AMI-ICH admissions had longer hospital length of stay (10.0 ± 13.6 vs. 5.1 ± 5.8 days), higher hospitalization costs ($106,300 ± 194,300 vs. 59,800 ± 77,500), and higher rates of percutaneous endoscopic gastrostomy (66.7 vs. 4.9%), but lower rates of tracheostomy (3.0% vs. 3.9%) and fewer discharges to home (21.5% vs. 62.6%) (all *p* < 0.001).

In the sub-group of AMI admissions with ICH that survived in-hospital stay (*n* = 13,689), 81.3% had a poor functional outcome at discharge. Female sex, White race, STEMI, use of fibrinolytic therapy, pulmonary artery catheterization, and mechanical circulatory support were associated with poor outcomes (Supplementary Table S3.). The 18-year temporal trends of poor functional outcomes showed a comparable trend in both STEMI and NSTEMI admissions (Figure 1C), with a slight increase in poor functional outcomes over the study period (Figure 1D).

## 4. Discussion

In this large cohort study, we noted ICH to complicate 0.2% of all AMI. ICH complicating AMI was associated with significant in-hospital mortality, greater resource utilization, and fewer discharges to home. Average AMI admissions complicated by ICH had hospitalization costs in excess of $100,000 despite lower rates of coronary interventions in these patients. STEMI presentation, acute organ failure, atrial fibrillation/flutter, use of fibrinolytic therapy, and mechanical circulatory support were significant predictors of ICH. These admissions received less-frequent coronary angiography, PCI, and CABG. Among ICH survivors, 81% had poor functional outcomes at discharge.

Consistent with existing evidence, ICH was noted more commonly in STEMI patients in this study [4,7]. Several studies have attributed the higher risk of ICH in STEMI to the use of fibrinolytic therapy [3,7]. Despite the decrease in thrombolysis, the concurrent increase in the use of dual and triple antiplatelet therapies could potentially explain the stable trend in ICH prevalence [11,19]. Furthermore, we identified a higher rate of atrial arrhythmias in admissions with ICH, and these patients remain at high risk due to the use of triple therapy, though there has been a trends towards dual therapy (i.e., eliminating aspirin) in these patients. Studies have identified a higher bleeding risk leading to ICH with antiplatelet therapy in these patients and advocated clinical judgment prior to prescribing these therapies [20]. Additionally, our study identified important predictors of ICH, such as the severity of illness, acute organ failure, and use of mechanical circulatory support. The use of mechanical circulatory support devices is associated with hemostasis and thrombosis due to cannulation in addition to thrombocytopenia due to transfusions and hemorrhage [17,21]. The use of anticoagulation with these devices further adds to the risk of ICH, and studies have shown up to 5% increase of ICH with their use in AMI [17].

Although challenging, the burden of ICH measured in terms of objective and subjective functional recovery using hospital-based outcomes is crucial to understanding their post-hospital course [10]. Discharge disposition along with the use of potential palliative measures like gastrostomy and/or tracheostomy have been recommended as measures of poor functional outcome in administrative databases [9]. Using these measures, we identified that 80% of those surviving hospital stay have poor functional outcomes, alluding to continued interaction with the health care system post-discharge and decreased quality of life. There was an increasing trend in the proportion of poor functional outcomes associated with AMI-ICH admissions. These appear to correlate with a decline in in-hospital mortality, suggesting that modern management strategies, while enabling survival of these morbid patients, are unable to improve the overall outlook. Therefore, as the complexities of the AMI population evolve, it is important to evaluate and manage the bleeding risk in these patients, specifically catastrophic events such as ICH.

### Limitations

Despite the HCUP-NIS database’s attempts to mitigate potential errors by using internal and external quality control measures, this study has several limitations. Echocardiographic data, angiographic variables, hemodynamic parameters, cerebral imaging, timing of ICH relative to AMI presentation, other in-hospital events and cardiac procedures, and reasons for not receiving aggressive medical care were unavailable in this database, which limits granularity and physiological assessments of disease severity. Information on therapies like anticoagulants and antiplatelets is also unavailable in the NIS database. The measure of disability used in this study would not capture post-discharge events. Additionally, ICH in our study could be a result of therapies used for AMI, and our data are only reflective of in-hospital outcomes. The HCUP-NIS cannot track patients across hospital admissions, and therefore the details of each admission are limited to the index hospitalization. Despite these limitations, this study provides contemporary knowledge highlighting the national temporal trends and burden of ICH in AMI population.

## 5. Conclusions

ICH is a rare complication in AMI admissions, but is associated with significantly higher mortality and resource utilization. A large proportion of AMI admissions with ICH appear to be further burdened by moderate to severe disability, as indicated by their short-term functional outcomes.

## Figures and Tables

**Table 1:** Baseline and in-hospital characteristics of AMI admissions with and without ICH.

Characteristic		ICH (*N* = 23,422)	No ICH (*N* = 11,599,106)	*p*
Age (Years)		71.6 ± 12.7	67.6 ± 14.2	<0.001
Female Sex		45.5	39.7	<0.001
Race	White	58.1	63.6	<0.001
	Black	9.7	7.9	
	Others ^a^	32.2	28.4	
Primary payer	Medicare	68.8	57.6	<0.001
	Medicaid	6.5	6.1	
	Private	18.2	27.9	
	Others ^b^	6.5	8.4	
Quartile of median household income for zip code	0–25th	23.9	24.4	<0.001
	26th–50th	27.5	27.2	
	51st–75th	23.5	24.5	
	75th–100th	25.2	23.9	
Charlson Comorbidity Index	0–3	12.3	37.6	<0.001
	4–6	49.2	44.5	
	≥7	38.4	17.9	
Comorbidities	Hypertension	58.0	62.5	<0.001
	Hyperlipidemia	28.7	47.5	<0.001
	Chronic kidney disease	11.8	11.0	<0.001
	Heart failure	31.8	29.3	<0.001
	Cancer	7.8	7.4	0.01
Hospital teaching status and location	Rural	8.5	11.2	<0.001
	Urban non-teaching	36.6	39.5	
	Urban teaching	54.9	49.3	
Hospital bed-size	Small	9.6	11.2	<0.001
	Medium	24.2	25.5	
	Large	66.2	63.3	
Hospital region	Northeast	19.7	19.6	<0.001
	Midwest	21.2	22.9	
	South	39.1	40.1	
	West	20.0	17.4	
AMI type	STEMI	51.9	37.1	<0.001
	NSTEMI	48.1	62.9	<0.001
Atrial fibrillation or flutter		24.3	17.4	<0.001
Supraventricular tachycardia		1.2	0.9	0.001
Ventricular tachycardia/fibrillation		13.8	8.0	<0.001
Multi-organ failure		30.8	9.4	<0.001
Cardiac arrest		16.0	5.0	<0.001
Cardiogenic shock		12.3	4.8	<0.001
Fibrinolytic therapy ^c^		8.4	2.2	<0.001
Coronary angiography		40.5	63.6	<0.001
Percutaneous coronary intervention		25.4	41.5	<0.001
Coronary artery bypass grafting		5.4	9.2	<0.001
MCS	Total	8.9	4.8	<0.001
	IABP	7.8	4.5	<0.001
	Percutaneous MCS	0.9	0.2	<0.001
	ECMO	0.6	0.0	<0.001
Pulmonary artery catheterization		1.9	1.1	<0.001
Invasive mechanical ventilation		36.4	5.9	<0.001
Acute hemodialysis		1.4	0.6	<0.001

Legend: Represented as percentage or mean ± standard deviation; ^a^ Hispanic, Asian or Pacific Islander, Native American, Others; ^b^ Self-Pay, No Charge, Others; ^c^ Only available from 2008 onwards. Abbreviations: AMI: acute myocardial infarction; ECMO: extracorporeal membrane oxygenation; IABP: intra-aortic balloon pump; ICH: intracerebral hemorrhage; MCS: mechanical circulatory support; NSTEMI: non-ST-segment-elevation myocardial infarction; STEMI: ST-segment-elevation myocardial infarction.

**Table 2:** Predictors of ICH in AMI admissions.

Total Cohort (*N* = 11,622,528)		Odds Ratio	95% Confidence Interval		*p*
			Lower Limit	Upper Limit	
Age (years)	≤75 years	Reference category			
	>75 years	0.71	0.69	0.73	<0.001
Female sex		1.08	1.05	1.11	<0.001
Race	White	Reference category			
	Black	1.23	1.18	1.29	<0.001
	Others ^a^	1.26	1.23	1.30	<0.001
Primary payer	Medicare	Reference category			
	Medicaid	1.36	1.29	1.44	<0.001
	Private	1.12	1.08	1.17	<0.001
	Others ^b^	1.31	1.24	1.39	<0.001
Charlson Comorbidity Index	0–3	Reference category			
	4–6	3.89	3.72	4.07	<0.001
	≥7	7.92	7.53	8.34	<0.001
Hypertension		0.81	0.79	0.84	<0.001
Hospital teaching status and location	Rural	Reference category			
	Urban non-teaching	1.60	1.48	1.64	<0.001
	Urban teaching	2.30	2.19	2.42	<0.001
Hospital bed-size	Small	Reference category			
	Medium	1.19	1.14	1.26	<0.001
	Large	1.53	1.47	1.60	<0.001
Hospital region	Northeast	Reference category			
	Midwest	0.97	0.93	1.01	0.22
	South	1.10	1.06	1.14	<0.001
	West	1.10	1.06	1.15	<0.001
AMI type	STEMI	Reference category			
	NSTEMI	0.46	0.45	0.47	<0.001
Cardiac arrest		1.82	1.74	1.90	<0.001
Cardiogenic shock		0.79	0.75	0.83	<0.001
Atrial fibrillation or flutter		1.11	1.08	1.15	<0.001
Supraventricular tachycardia		1.00	0.89	1.13	0.94
Ventricular tachycardia/fibrillation		1.03	0.98	1.07	0.22
Fibrinolytic therapy ^c^		4.28	4.07	4.49	<0.001
Coronary angiography		0.53	0.52	0.55	<0.001
Percutaneous coronary intervention		0.67	0.65	0.70	<0.001
Coronary artery bypass grafting		0.46	0.43	0.49	<0.001
Pulmonary artery catheterization		0.97	0.88	1.07	0.59
Mechanical circulatory support		1.61	1.52	1.70	<0.001
Multi-organ failure		2.48	2.40	2.56	<0.001

Legend: ^a^ Hispanic, Asian or Pacific Islander, Native American, Others; ^b^ Self-Pay, No Charge, Others; ^c^ Only available from 2008 onwards. Abbreviations: AMI: acute myocardial infarction; ICH: intracerebral hemorrhage.

## Supplementary Materials

The following are available online at https://www.mdpi.com/2077-0383/9/9/2717/s1, Table S1: Administrative codes used for identification of diagnoses and procedures, Table S2: Multivariable regression for in-hospital mortality in AMI, Table S3: Predictors of poor functional outcomes in AMI-ICH survivors.

## Abbreviations

AMI	Acute myocardial infarction
CABG	Coronary artery bypass grafting
HCUP	Healthcare cost and utilization project
ICD-9CM	International Classification of Diseases-9 Clinical Modification
ICD-10CM	International Classification of Diseases-10 Clinical Modification
ICH	Intracranial hemorrhage
NIS	National/nationwide inpatient sample
NSTEMI	non-ST-segment elevation myocardial infarction
PCI	Percutaneous coronary intervention
STEMI	ST-segment elevation myocardial infarction
