# Supplementary material for: Intracranial Hemorrhage Complicating Acute Myocardial Infarction: An 18-Year National Study of Temporal Trends, Predictors, and Outcomes
Source: J Clin Med. 2020 Aug 22;9(9):2717. doi: 10.3390/jcm9092717 (PMC7565584; doi:10.3390/jcm9092717)
Supplement: Supplementary file 1 [file jcm-09-02717-s001.pdf]

Supplementary Table S1. Administrative codes used for identification of diagnoses and procedures.

Comorbidity	International Classification of Diseases 9.0 Clinical Modification Codes
Cardiac arrest	427.5, 427.4, 427.41, 427.42, 99.60, 99.63
Coronary angiography	37.22, 37.23, 88.53-88.56
Percutaneous coronary intervention	00.66, 36.01, 36.02, 36.05, 36.06, 36.07, 88.57
Invasive hemodynamic assessment	89.63, 89.64, 89.66, 89.67, 89.68
Mechanical circulatory support	37.61, 37.68, 39.65
Invasive mechanical ventilation	96.7, 96.70, 96.71, 96.72
Hemodialysis	39.95
Multi-organ failure	570.0, 572.2, 573.3, 573.4
	518.81, 518.82, 518.85, 786.09, 799.1, 96.7, 96.70, 96.71, 96.72
	584, 584.5, 584.6, 584.7, 584.8, 584.9
	286.6-286.9, 287.4, 287.5
	293, 293.0, 293.1, 293.8, 293.81-293.84, 293.89, 293.9, 348.1, 348.3, 348.30, 348.81, 348.39, 780.01, 780.09, 89.14
Tracheostomy	311, 312, 3121, 3129
Percutaneous endoscopic gastrostomy	430, 431, 4311, 4319, 4432, 4438, 4439
Fibrinolytic usage	V45.88, 99.10

Supplementary Table S2. Multivariable regression for in-hospital mortality in AMI.

Total cohort (N=11,622,528)		Odds ratio	95% confidence interval		*P*
			Lower Limit	Upper Limit	
**Intracranial hemorrhage**		5.60	5.42	5.79	<0.001
**Age (years)**	**≤75 years**	Reference category			
	**>75 years**	2.06	2.04	2.07	<0.001
**Female sex**		1.14	1.13	1.14	<0.001
**Race**	**White**	Reference category			
	**Black**	0.87	0.86	0.88	<0.001
	**Others**	1.08	1.07	1.08	<0.001
**Primary payer**	**Medicare**	Reference category			
	**Medicaid**	0.78	0.77	0.79	<0.001
	**Private**	0.68	0.67	0.69	<0.001
	**Others**	0.95	0.93	0.96	<0.001
**Hospital teaching status and location**	**Rural**	Reference category			
	**Urban Non-Teaching**	1.06	1.05	1.07	<0.001
	**Urban Teaching**	1.14	1.13	1.15	<0.001
**Hospital bed-size**	**Small**	Reference category			
	**Medium**	1.09	1.08	1.10	<0.001
	**Large**	1.23	1.22	1.24	<0.001
**Hospital region**	**Northeast**	Reference category			
	**Midwest**	0.99	0.98	0.99	<0.001
	**South**	1.06	1.05	1.06	<0.001
	**West**	0.85	0.85	0.86	<0.001
**Charlson Comorbidity**	**0-3**	Reference category			

**Index**	**4-6**	2.19	2.17	2.21	<0.001
	**≥ 7**	2.63	2.60	2.66	<0.001
**Hypertension**		0.68	0.68	0.69	<0.001
**Type of AMI**	**ST-segment elevation**	Reference category			
	**Non-ST-segment elevation**	0.40	0.40	0.40	<0.001
**Cardiogenic shock**		2.96	2.94	2.99	<0.001
**Cardiac arrest**		13.04	12.91	13.17	<0.01
**Atrial fibrillation/flutter**		1.09	1.08	1.10	<0.001
**Supraventricular tachycardia**		0.76	0.74	0.78	<0.001
**Ventricular fibrillation/tachycardia**		0.58	0.57	0.58	<0.001
**Multi-organ failure**		2.83	2.81	2.86	<0.001
**Coronary angiography**		0.37	0.36	0.37	<0.001
**Fibrinolytic use**		0.59	0.58	0.61	<0.001
**Percutaneous coronary intervention**		0.40	0.40	0.41	<0.001
**Coronary artery bypass grafting**		0.45	0.45	0.46	<0.001
**Pulmonary artery catheterization**		1.31	1.28	1.33	<0.001
**Mechanical circulatory support**		2.06	2.03	2.08	<0.001
**Invasive mechanical ventilation**		3.14	3.11	3.17	<0.001
**Acute hemodialysis**		1.92	1.88	1.96	<0.001
**Vascular complications**		1.22	1.19	1.26	<0.001

Abbreviations: AMI: acute myocardial infarction

Supplementary Table S3. Predictors of poor functional outcomes in AMI-ICH survivors.

Total cohort (N=13,689)		Odds ratio	95% confidence interval		*P*
			Lower Limit	Upper Limit	
**Age (years)**	**≤75 years**	Reference category			
	**>75 years**	1.05	0.95	1.17	0.34
**Female sex**		1.28	1.17	1.40	<0.001
**Race**	**White**	Reference category			
	**Black**	0.71	0.62	0.82	<0.001
	**Others**	0.99	0.89	1.09	0.84
**Primary payer**	**Medicare**	Reference category			
	**Medicaid**	0.74	0.62	0.88	0.001
	**Private**	0.87	0.77	0.98	0.03
	**Others**	0.86	0.72	1.03	0.11
**Hospital teaching status and location**	**Rural**	Reference category			
	**Urban Non-Teaching**	1.51	1.28	1.78	<0.001
	**Urban Teaching**	1.40	1.19	1.64	<0.001
**Hospital bed-size**	**Small**	Reference category			
	**Medium**	1.46	1.25	1.69	<0.001
	**Large**	1.80	1.57	2.06	<0.001
**Hospital region**	**Northeast**	Reference category			
	**Midwest**	0.75	0.65	0.88	<0.001
	**South**	0.62	0.54	0.71	<0.001
	**West**	0.52	0.45	0.61	0.001
**Charlson Comorbidity Index**	**0-3**	Reference category			
	**4-6**	0.74	0.64	0.86	<0.001

	≥ 7	1.03	0.87	1.22	0.76
**Type of AMI**	**ST-segment elevation**	Reference category			
	**Non-ST-segment elevation**	0.69	0.63	0.77	<0.001
**Cardiogenic shock**		0.94	0.77	1.15	<0.57
**Cardiac arrest**		0.76	0.62	0.92	0.004
**Atrial fibrillation/flutter**		0.93	0.84	1.03	0.16
**Supraventricular tachycardia**		1.50	0.96	2.33	0.07
**Ventricular fibrillation/tachycardia**		1.07	0.91	1.27	0.41
**Coronary angiography**		0.73	0.64	0.82	<0.001
**Fibrinolytic use**		1.54	1.25	1.90	<0.001
**Percutaneous coronary intervention**		0.91	0.80	1.04	0.19
**Coronary artery bypass grafting**		1.06	0.87	1.29	0.58
**Pulmonary artery catheterization**		2.68	1.56	4.60	<0.001
**Mechanical circulatory support**		1.81	1.43	2.29	<0.001
**Invasive mechanical ventilation**		0.95	0.84	1.07	0.39
**Acute hemodialysis**		1.11	0.72	1.70	0.63
**Multi-organ failure**		0.99	0.87	1.12	0.84
**Vascular complications**		2.38	1.41	3.99	0.001

Abbreviations: AMI: acute myocardial infarction; ICH: intracerebral hemorrhage
